# Hoarseness of Voice Following Left Supraclavicular Brachial Plexus Block: A Case Report

**DOI:** 10.31729/jnma.7087

**Published:** 2021-09-30

**Authors:** Subin Shrestha, Sadikshya Regmi, Gopendra Deo, Indra Narayan Shrestha

**Affiliations:** 1 Department of Anaesthesia and Critical Care, Chitwan Medical College Teaching Hospital, Bharatpur, Nepal

**Keywords:** *brachial plexus block*, *hoarseness*, *recurrent laryngeal nerve*

## Abstract

Supraclavicular brachial plexus block is extensively used for primary regional anaesthesia as well as postoperative analgesia for the surgical procedures of the upper limb. The evidence for the use of ultrasound in supraclavicular brachial plexus is growing day by day as it has the advantage of allowing real time visualisation of the plexus, pleura and vessels along with the needle and local anaesthetics spread. Despite this, complications can even arise with ultrasound guided supraclavicular brachial plexus block. Hoarseness of voice due to recurrent laryngeal nerve block is a rare complication of supraclavicular brachial plexus block. There are few reported cases of hoarseness of voice following the right supraclavicular block. There is only one reported case of hoarseness of voice following the left supraclavicular block. Here, we report a case of a 16-year-old boy who developed hoarseness of voice due to left recurrent laryngeal nerve following ultrasound guided left supraclavicular brachial plexus block.

## INTRODUCTION

Supraclavicular brachial plexus block is an excellent option for the surgical procedure from the midhumerus to the fingertips. It is associated with various complications such as pneumothorax, arterial puncture, hematoma, diaphragmatic palsy, local anaesthesia systemic toxicity (LAST) and hoarseness of voice.^1^ The use of ultrasound has improved the success rate of the block with excellent localisation and safety margin.^2^ However, ultrasound can create a false sense of security. Hence, complications can arise even with the ultrasound guided blocks. Hoarseness of voice due to recurrent laryngeal nerve block (RLN) is a rare complication of supraclavicular block. It has been reported in the right supraclavicular block and in an interscalene approach. Here, we present a case report of hoarseness of voice following ultrasound guided left supraclavicular block.

## CASE REPORT

A 16-year-old boy, American Society of Anesthesiology (ASA) grade I, weighing 52kg, was planned for implant removal of united fracture radius and ulna with plate in situ under ultrasound guided supraclavicular brachial plexus block. No abnormalities were detected in preoperative evaluation. All the preoperative investigations were unremarkable. The procedure was explained to the patient and the parents. Anaesthesia consent was taken from parents. He was shifted to the operation room and the standard ASA monitoring was done. His pulse rate was 105 bpm, BP was 110/70 mm of Hg and oxygen saturation was 100% on room air. IV access was secured with 20G cannula in the contralateral arm.

Under strict aseptic precaution, high frequency linear transducer probe of Siemens Acuson Ultrasound machine was placed on supraclavicular fossa in the transverse plane just above the clavicle. Fine adjustments were made to perfectly visualise the subclavian artery and brachial plexus. Skin was infiltrated with 1ml local anaesthetic using 27G 1ml needle at the presumed site of needle insertion. Brachial plexus was blocked using 22G, 5mm stimuplex ultra needle with in-plane technique using 20ml of 0.25% Ropivacaine and 15ml of 0.25% Bupivacaine after repeated negative aspiration. After a few minutes, the patient complained of difficulty in speaking. The change in his voice was also noticed which was not present before. He became very anxious and also complained of difficulty in breathing.

**Figure 1:**
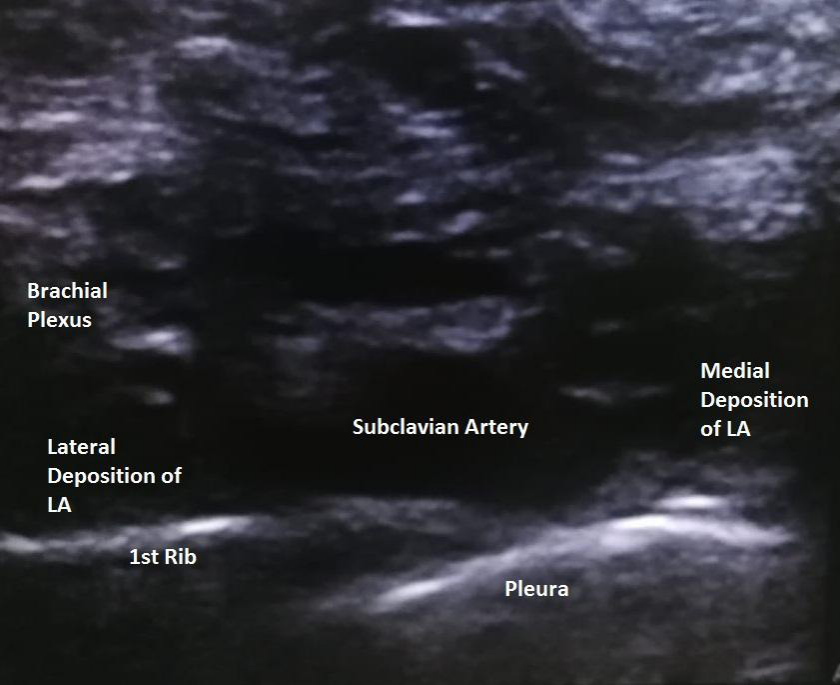
Ultrasound image after block.

We checked for the adverse effects of local anaesthesia systemic toxicity like tongue or perioral numbness, light headedness, tinnitus, visual and auditory disturbances but none were present. Then we examined the respiratory system to look for any signs and symptoms of pneumothorax. However, respiratory examination was normal and oxygen saturation was 100% with 5 litres of oxygen via face mask. Assessment of effect of brachial plexus blockade was done and complete sensory and motor blockade was achieved. Midazolam 1mg was given to relieve the anxiety. Despite this, the patient became more restless, agitated and tachypnoeic. Hence, it was decided to intubate the patient and put on mechanical ventilation. Induction was done with inj. Propofol (2mg/kg) and endotracheal intubation was facilitated with injection rocuronium (0.6mg/kg). Airway was secured with a 7.0mm endotracheal tube. Immediately after intubation, ultrasound scan of the neck region was performed. There was anechoic area lateral to the subclavian artery probably due to the deposition of local anaesthetics A similar anechoic area just medial to the subclavian artery, probably indicating the deposition of either local anaesthetics or accumulation of blood was also seen (Figure 1).

Left RLN block was considered as our differential diagnosis. Similar ultrasound image and clinical scenario were also described by Naz, et al.^3^ which corroborates the suspicion of our diagnosis. In addition to this, ultrasound scan of the lungs was performed to see lung sliding and subsequently rule out pneumothorax.

**Figure 2:**
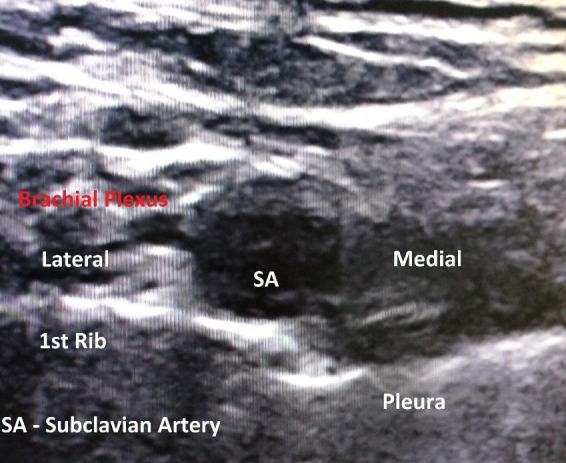
Ultrasound image 24 hours after block.

The patient was hemodynamically stable, bilateral equal air entry was present and oxygen saturation was 100%. Hence, the surgery was resumed. Intraoperative period was uneventful and inj. Dexamethasone 8mg was given. The surgery was completed in 2 hours. During extubation, vocal cord was assessed with a flexible scope (AmbuScope). After administration of the reversal agent, AmbuScope was inserted through the endotracheal tube and the endotracheal tube was slowly pulled until the vocal cord was visualised. Disparity in the movement of the vocal cord was seen with left moving less compared to right. Unfortunately, we could not further assess the vocal cord as the patient was awake and it was causing him discomfort. Hence, the patient was extubated. Residual hoarseness of voice was still present, but he was more comfortable and calmer. The patient was reassessed after 4 hours. He was allowed to take sips of water, but he coughed indicating aspiration due to persistence of vocal cord abnormality. He was kept nil per oral for 8 hours, after which this problem subsided. On the next day, repeat ultrasound scan of the neck region was done which showed absence of anechoic area on the medial and lateral of the subclavian artery (Figure 2).

## DISCUSSION

The complication that may arise due to ultrasound guided brachial plexus block depends on the approaches to blocking the brachial plexus. For instance, diaphragmatic paralysis is common with the interscalene approach but is absent with the axillary approach. There is evidence that the use of ultrasound reduces the incidence of complications like pneumothorax and LAST.^4^ Although it is considered rare to have a complication in experienced hands and with the use of ultrasound, there are a few reports of complication in spite of all these. One of such rare complications is hoarseness of voice due to RLN block. This is an occasional complication of the interscalene brachial plexus block, but it is quite rare following supraclavicular approach. The incidence of hoarseness of voice following supraclavicular brachial plexus using the landmark technique is 1.3%.^5^ We do not have any data on RLN block with USG guided supraclavicular block.

RLN block is more common in the right supraclavicular block in comparison to left supraclavicular block due to anatomic consideration.^3^ Right RLN is close to the right brachial plexus as it hooks around the right subclavian artery. On the left side, the left vagus nerve runs close to the left brachial plexus and left RLN winds around the aortic arch and runs very close to trachea and esophagus. There are few reported cases of RLN block following the right supraclavicular block. Gupta et al and Sahu et al reported hoarseness of voice following ultrasound guided right supraclavicular block.^6,7^ Similarly, Balaji et al. reported RLN block and Horner's syndrome in a same patient following peripheral nerve stimulator (PNS) guided right supraclavicular block.^8^ The RLN block due to left supraclavicular block is very rare. There is only one case report of an RLN block following the left supraclavicular block. Naz, et al. reported RLN block following left supraclavicular block.^3^

Most of the previous literature has attributed the hoarseness of voice to excessive local anaesthetics spread to RLN. In order to understand this, it is very essential to have an understanding of the tissue surrounding the brachial plexus. The exact anatomy of the tissues surrounding the brachial plexus is not clearly understood and there still remains controversy about whether a fibrous sheath or rigid anatomical tunnel exists around brachial plexus.^9^ One viewpoint is that brachial plexus is enclosed in a connective tissue-based sheath which is a continuation of the prevertebral fascia that covers and surrounds the scalene muscles. It continues to axilla in a neurovascular bundle containing axillary artery, axillary vein and the median, ulnar and radial terminal nerves.^10^ Another viewpoint suggests that there may not be 'sheath' covering the brachial plexus, instead brachial plexus lies in the tissue plane between rigid anatomical structures acting as a tunnel.^11^ However, both an enveloping fibrous sheath and rigid anatomical tunnel are consistent with the clinical observation that local anaesthetics is more likely to spread longitudinally along the nerve rather than circumferentially. Local anaesthetics spread either distally or proximally along the nerve in the longitudinal axis.^9^

If the brachial plexus was considered to be covered with fibrous sheath, RLN block would not have been caused by proximal and longitudinal spread of local anaesthetics as RLN is anterior and outside the fascial sheath covering brachial plexus.^3^ In this case, the drug might have been deposited more medial and outside of the brachial plexus sheath due to incorrect needle placement. Similarly, if brachial plexus was considered to be enclosed in a rigid anatomical tunnel without sheath, RLN block would have caused medial spread of local anaesthetics which may result from the use of large volume of local anaesthetics. Unilateral RLN is not of much clinical significance other than annoyance to the patient. However, it can cause complete airway obstruction in a patient with a pre-existing contralateral RLN palsy due to previous neck surgery.^12^

In our case, there was a block of left vagus nerve, which contained the fibre of left RLN. Hence, it is very essential to visualise needle tip as well as spread of local anaesthetics throughout the procedure. Similarly, we should also aim for lower volumes of local anaesthetics when we are performing under ultrasound guidance.
